# Effects of Saikosaponin D on Apoptosis, Autophagy, and Morphological Structure of Intestinal Cells of Cajal with Functional Dyspepsia

**DOI:** 10.2174/0113862073262404231004053116

**Published:** 2023-10-10

**Authors:** Yi Zeng, Li Zhou, Ying Wan, Ting Fu, Paidi Xu, Hongxing Zhang, Ying Guan

**Affiliations:** 1 Department of Hospital Infection Management Office, Wuhan Hospital of Integrated Traditional Chinese and Western Medicine, Wuhan, China;; 2 Department of Rehabilitation, Wuhan Hospital of Integrated Traditional Chinese and Western Medicine, Wuhan, China;; 3 Department of Gastroenterology, Wuhan Hospital of Integrated Traditional Chinese and Western Medicine, Wuhan, China;; 4 Department of Traditional Chinese Medicine, Wuhan Hospital of Integrated Traditional Chinese and Western Medicine, Wuhan, China;; 5 College of Acupuncture and Orthopedics, Hubei University of Chinese Medicine, China;; 6 Jianghan University, Wuhan, China

**Keywords:** Saikosaponin D, functional dyspepsia, autophagy, gastrointestinal motility, ICCs, apoptosis

## Abstract

**Objective:**

Functional dyspepsia (FD) is one of the most common gastrointestinal diseases, with a global prevalence of 10%-30%. However, the specific pathogenesis of FD has not yet been determined. As such, the aim of this study was to investigate the effects of saikosaponin D (SSD) administration on the apoptosis, autophagy, and morphological structure of the intestinal cells of Cajal (ICCs) in FD.

**Methods:**

A rat model of FD was constructed by stimulating the rat tail with a sponge clamp at one-third of the distal tail length. An autophagy model was constructed for ICCs using glutamate. The apoptosis rate in each group of cells was determined using flow cytometry. The expressions of ghrelin and substance P (SP) were detected using ELISA.

**Results:**

The body weight and food intake of male and female rats in the SSD group were consistently higher than those in the model group. The SSD group showed substantial improvement compared with the model group, with no inflammatory cell infiltration and normal gastric mucosal structures. After intervention with SSD, the ultrastructure of the ICCs considerably improved and was clear. Compared with the model group, the expressions of LC3 I/II, ghrelin, and SP proteins in the SSD group were significantly upregulated, and the apoptosis rate was significantly reduced.

**Conclusion:**

The administration of SSD improved ICC morphology and structure, inhibited excessive autophagy, and improved FD, a gastrointestinal motility disorder, by regulating ghrelin and SP levels.

## 1 INTRODUCTION

Functional dyspepsia (FD) is a common clinical digestive disorder that is characterized by abdominal pain and burning, postprandial bloating, early satiety, and vomiting [1]. The pathogenesis of FD may be related to hypersensitivity of the stomach and duodenum, gastrointestinal motility disorders, *Helicobacter pylori* infection, and psychosomatic factors [2]. Epidemiological data show that the worldwide prevalence of indigestion in the population is 10%-30% [3]. FD symptoms are often recurrent and persistent, seriously affecting the quality of life of patients. FD is a common disease that can seriously endanger human health. The incidence of FD is increasing in China, so the disease and associated risks cannot be ignored.

Gastrointestinal motility disorders are an important pathophysiological basis of FD [4]. The enteric nervous system can independently regulate intestinal function, including important enteric neurotransmitters such as substance P (SP), nitric oxide (NO), and ghrelin. Ghrelin is an important gastrointestinal hormone that is currently the only endogenous ligand for growth hormone secretagogue receptors (GHSRs). Ghrelin, also known as gastrodin-related peptide, controls the appetite, stimulates the release of growth hormones, promotes gastric emptying, and enhances gastric motility [5, 6]. Substance P (SP) has an inhibitory effect on mucus secretion from the gastrointestinal glands, thereby stimulating intestinal motility [7-9]. A meta-analysis revealed abnormal intestinal mucosa and immune response in FD patients, and inflammatory factors such as IL-6 and IL-1β were associated with the onset and duration of FD symptoms [10]. The clinical use of progastric drugs such as morpholine and mosapride is effective in treating FD, but the long-term use of these drugs can have adverse side effects on the liver, kidney, and gastrointestinal function [11]. Some herbs, such as peppermint leaves, licorice, and calamus root, can be used to treat functional gastrointestinal disorders, such as FD [12]; as such, more effective active ingredients and herbal preparations should be developed from these herbs for the treatment of FD.

The intestinal cells of Cajal (ICCs) are pacemaker cells in the gastrointestinal tract that regulate intestinal motility [13]. The loss of ICCs leads to gastrointestinal motility disorders [14]. Autophagy is an intracellular homeostatic mechanism that plays an important role in the pathogenesis of gastrointestinal diseases [15], and excessive autophagy of ICCs may be one of the mechanisms underlying the development of gastrointestinal motility disorders such as FD. Many herbs can improve gastrointestinal tract function by modulating the function of ICCs and the autophagic response. For example, Chai Hu is a herb commonly used to treat chronic inflammation, pain, and fever [16]. Additionally, saikosaponin D (SSD) is the main active monomer of saikosaponin, which has anti-inflammatory [17], antibacterial [16], and immunomodulatory effects [18]. However, the role of SSD in the glutamate-induced autophagy model of ICCs has not yet been determined. Therefore, the aim of this study was to investigate the effects of SSD on the ultrastructure and autophagy of ICCs in an autophagy model.

## 2 MATERIALS AND METHODS

### 2.1 Main Materials and Reagents

Chai Hu saponin D (S114050) and mosapride (M129673) were purchased from Aladdin. Eosin (C0109) and hematoxylin (C0107) were purchased from Biyuntian; epoxy resin 812 (90529-77-4) and lead nitrate (1101125) were purchased from SPI. LC3 I/II (PAB34117), GAPDH (PAB36269), goat antirabbit IgG (SAB43714), conjugated Alexa Fluor 488, Affinipure goat antirabbit (SAB43742), c-Kit (PAB36544), and LC-3II (PAB34117) were purchased from Bioswamp. Penicillin-streptomycin solution (P1400) was obtained from Solarbio. Collagenase II (17101015) was purchased from Gibco, and rat SCF (SRP3251) was purchased from Sigma-Aldrich. An Annexin V-FITC/PI Apoptosis Assay Kit (556547) was purchased from BD Biosciences. ELISA kits for RAT Ghrelin (RA20817) and RAT SP (RA20646) were obtained from Bioswamp.

### 2.2 Methods

#### 2.2.1 Construction of FD Rat Model

All animals were obtained from Three Gorges University. Twenty-four SD rats, weighing 250±20 g, male and female, were housed in specific pathogen-free (SPF) conditions. The rearing environment was maintained at 22-26°C, 50%-60% relative humidity, and 12 h each of artificial light and dark. The FD models were constructed based on previous experience [19]. The tails of the rats were stimulated with a sponge clamp at one-third of the distal tail length for 30 min each time, causing chronic stress without injury, and this was repeated four times per day (8:30, 10:30, 14:00, and 16:00) for 7 days. General status observations, including appetite, activity, weight, secretions, and hair condition, were recorded daily during the modeling period. The small intestinal propulsion rate in rats was determined using a nutritive semisolid paste gavage method.

#### 2.2.2 Animal Grouping and Drug Intervention

The SD rats were randomly divided into four groups of six rats each, half males and half females: control group (Control), model group (Model), SSD intervention group (SSD: 2 mg/kg/d [20], intraperitoneal injection), and positive drug group (PC: mosapride, 1.37 mg/kg/d, *via* gavage). The pharmacological intervention was started on the second day after the completion of modeling and administered continuously for 7 d. Equal doses of saline were administered to the blank and model groups of bacteria. On the 8th day, the rats were anesthetized with 3% pentobarbital sodium sacrificed, and gastric sinus tissue was collected.

#### 2.2.3 HE Staining

The gastric sinus tissues were dewatered and embedded in wax according to the standard procedure, with a slice thickness of 3 μm. The slices were gently washed with water for 1-2 min, then stained with hematoxylin for 3-6 mins, followed by staining with 1% hydrochloric acid alcohol for 1-3 s. The blue-promoting solution turned to blue in 5-10 s. Then, staining was performed with 0.5% eosin solution for 2-3 min, which was followed by rinsing with 80% ethanol for 15-30 s, then with 95% ethanol for 15-30 s, anhydrous ethanol for 1-2 s, xylene (I) for 2-3 s, and finally with xylene (II) for 2-3 s. Neutral tree sealant. Finally, the sample was photographed using a microscope, and images of the relevant parts of the samples were captured using a Leica Application Suite graphics system.

#### 2.2.4 Transmission Electron Microscopy

The samples were first placed in fresh fixative as quickly as possible. The tissue was cut into 1 mm^3^ pieces. Then, 2.5% glutaraldehyde was administered at a dose of at least 3 mL and fixed at 4°C for at least 30 min prior to detection. The cells were washed three times with phosphate-buffered saline (PBS; 0.1 mol/L PBS buffer for 10 min each). Then, 1% osmium acid was fixed for 1 h. The cells were washed three times for 10 min each in 0.1 mol/L PBS and then dehydrated. Semi-impregnation was performed with acetone at 40°C for 6 h. Pure epoxy resin 812 was fully impregnated at 40°C for 4 h. Subsequently, the embedding treatment was performed. The cells were sliced into 60 nm ultra-thin sections. Uranium acetate staining was performed for 20 min while avoiding light. The cells were then stained with lead citrate for 15 min in the dark. Finally, the cells were observed using transmission electron microscopy and photographed.

#### 2.2.5 Western Blotting

The gastric sinus tissue was cut into tiny pieces. Lysate was added to these pieces (200 μL of lysate per 20 mg of tissue), and protein concentration was determined using a BCA. The proteins were separated *via* SDS-PAGE electrophoresis with 20 μg of protein loaded per well, transferred to PVDF membranes, and kept overnight at 4°C with 5% skim milk powder. Primary antibodies against LC3 I/II (1:1000) and GAPDH (1:1000) were added and incubated with the membrane for 1 h at room temperature. The secondary antibody, goat anti-rabbit IgG (1:10,000), was added, and the membrane was incubated for 1 h at room temperature. After washing the membrane, the ECL chemiluminescence reagent was added, in full contact with the membrane, and placed in a fully automated chemiluminescence analyzer for detection. TANON GIS software was used to read the grayscale values of the protein bands.

#### 2.2.6 Primary ICC Isolation

Primary rat ICCs were isolated following the previously reported method [21]. After the rats were anesthetized with 3% sodium pentobarbital, the gastric sinus was dissected longitudinally, immediately incised along the lesser curvature of the stomach, and flushed with pre-chilled PBS (containing 2% of both antibodies). The gastric muscle strips were cut into small slices of approximately 1 × 1 mm and added to a type II collagenase digestion solution. The gastric tissue fragments were digested in a constant-temperature water bath at 37°C for approximately 50 min, which were then centrifuged at 1000 rpm for 5 min. The supernatant was then discarded, and the tissues were resuspended in PBS for 5 min. The tissues were again centrifuged; the supernatant was discarded, and the cells were resuspended in M199 complete medium and then passed through a 200-mesh sieve. An equal amount of Ficoll 400 density gradient solution was added to the cells, which were centrifuged at 1000 r/min for 7 min. Then, the cell density was adjusted to 1×10^9^/L, with 4 mL of inoculum for each 25 cm^2^ culture flask. The cells were then incubated in a 37°C CO_2_ incubator for 24 h. After this, the solution was changed, and the unadhered cells were flushed out. The solution was changed once every 3 d, and cells in logarithmic growth phase were used for the next experiment.

#### 2.2.7 Immunofluorescence Single Label

The samples from each group were washed with 1 mL of PBS to which 1 mL of 4% paraformaldehyde was added. Then, the cells were fixed at room temperature for 30 min. Next, 1 mL of 0.5% Triton X-100 was added, and the cells were permeabilized at room temperature for 30 min. Then,1 mL of 5% BSA was added, and the samples were closed for 1 h at 37°C. Following this, 300 μL of diluted antibody in PBS was added, and the samples were incubated overnight at 4°C. Then, the samples were washed 2 times with 1 mL of PBS, 300 μL of diluted antibody in PBS was added at 37°C, the samples were incubated for 1h, then washed twice with 1 mL of PBS. Next, 300 μL of antifluorescence quenching blocking solution (containing DAPI) was added. Finally, observations and photographs were obtained *via* fluorescence microscopy.

#### 2.2.8 Construction of ICC Autophagy Model

The cells were collected; the concentration of the cell suspension was adjusted with a complete medium and divided into 6-well plates, ay 5×10^5^ cells/well (2 mL per well), and incubated for 24 h at 37°C in a 5% CO_2_ incubator. The model group was stimulated with 5 mmol/L glutamate for 3 h to construct an ICC autophagy model [21, 22]. The incubation continued for 3 h at 37°C in a 5% CO_2_ incubator. The experiment was then divided into the following four groups: control group (primary ICCs), model group (5 mmol/L glutamate stimulation for 3 h), SSD group (5 mmol/L glutamate stimulation for 3 h followed by SSD action with 10 μmol/L for 48 h), and 3-MA (5 mmol/L glutamate stimulation for 3 h followed by 5 mmol/L [23] 3-MA treatment for 48 h) group. Follow-up tests were also performed.

#### 2.2.9 CCK8

The cells were collected, and the concentration of cell suspension was adjusted and divided into 96-well plates, with 3×10^3^ cells/well (100 µL per well), and incubated overnight at 37°C in a 5% CO_2_ incubator to adhere the cells to the wall. The cells were treated with the different groups and incubated for 3 h. The cell culture plates were removed, 10 μL of CCK8 solution was added to each well, and incubation was continued for 4 h. The cells were then incubated for 4 h. The absorbance of each well was measured at 450 nm using an ELISA plate reader.

### 2.3 Flow Cytometry

The cells of each group were collected *via* centrifuging at 400× g and 4°C for 5 min. Then, 1 mL of precooled PBS was added, the cells were mixed by gently blowing on them and then centrifuging at 400× g for 5 min at 4°C. The cells were then resuspended in 200 μL of PBS, 10 μL of Annexin V-FITC and 10 μL of PI were added, and the cells were incubated for 30 min at 4°C, protected from light. After this, 300 μL of PBS was added, and then flow-through detection was conducted. The analysis was performed using NovoExpress analysis software.

### 2.4 ELISA

The standards were diluted first. Standard, blank, and sample wells were set up on the enzyme-coated plate. A total of 50 μL of different concentrations of standards was added to the standard wells in turn. The sample wells were first spiked with 40 μL of sample; then, 10 μL of biotin-labeled antibody was added. Except for the blank wells, 50 μL of enzyme standard reagent was added to each well, and the plates were sealed with sealing film and incubated at 37°C for 30 min. After washing, color was developed by adding 50 μL of color developer A and then 50 μL of color developer B to each well, gently shaking and mixing, then waiting for 10 min at 37°C while the wells were protected from light. Finally, the reaction was terminated by adding 50 μL of termination solution to each well. The absorbance (OD) of each well was measured sequentially at 450 nm, with blank wells adjusted to zero.

### 2.5 Statistical Analysis of Data

Statistical analysis was performed using SPSS 19.0, and the results are expressed as mean ± standard deviation (SD); one-way ANOVA was used for comparison between multiple groups, and a t-test was used for comparison between groups, with *P<*0.05 indicating a statistically significant difference.

## 3 RESULTS

### 3.1 Construction of FD Rat Model

**Fig. (1):**
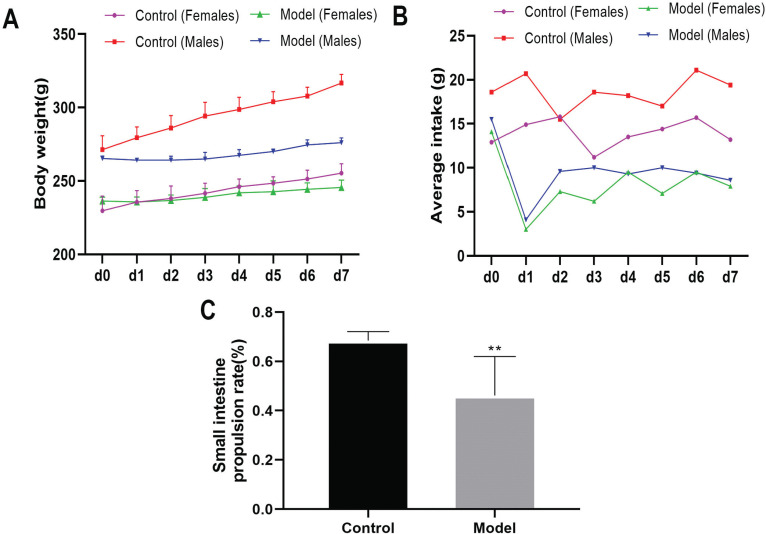
Model construction. Changes in (**A**) body weight, (**B**) food intake, and (**C**) small intestinal propulsion rate of rats.

General status observations, including appetite, activity, weight, secretions, and hair condition, were recorded daily during the modeling period. The body weight and food intake of both male and female rats in the model group were consistently lower than those in the control group (Fig. **1A** and **B**). The small intestine propulsion rate of the rats in the model group was significantly lower than that in the control group (*P<*0.01, Fig. **1C**). In addition, the rats in the model group showed symptoms such as fluid excretion and reduced activity. This result indicated that the FD rat model was successfully constructed.

### 3.2 Effect of SSD on Pathological Changes and Ultrastructure of ICCs in FD Rat Model

**Fig. (2):**
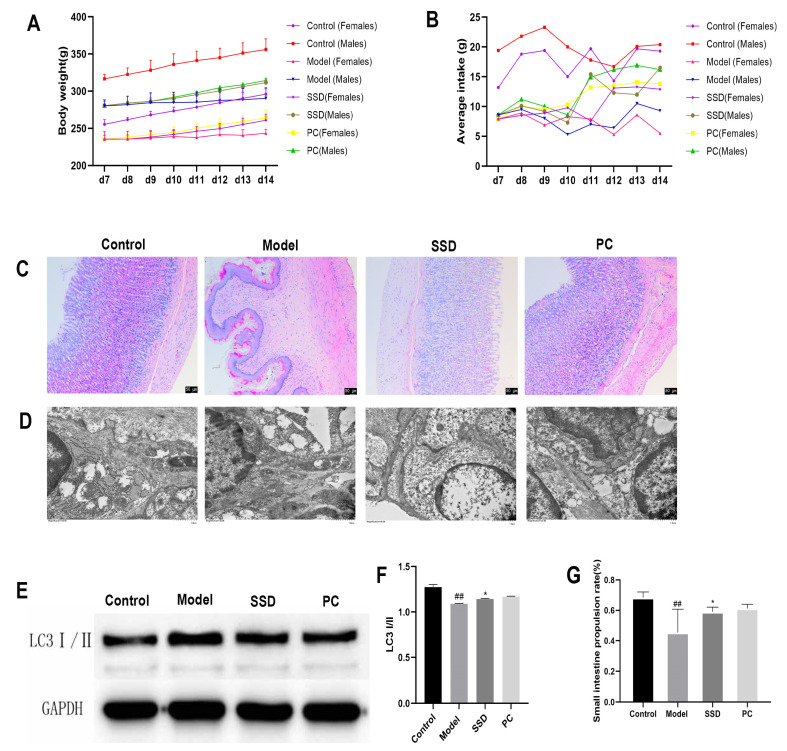
Effect of SSD administration on pathological changes and ultrastructure of ICCs in FD rat model. Changes in (**A**) body weight and (**B**) food intake of FD rats. (**C**) HE staining used to observe pathological morphology of gastric sinus tissue. (**D**) Electron microscopy conducted to observe ultrastructure of ICCs in gastric sinus tissue. (**E**) Detection of apoptosis in gastric antrum tissue through TUNEL assay. (**F**) Western blotting results demonstrating expression of autophagy-related protein LC3 II/I in gastric sinus tissue. (**G**) Small intestinal propulsion rate in rats.

Next, we administered pharmacological interventions to the rats in the experimental groups. The general health status of the rats was also observed. As shown in Fig. (**2A** and **B**), the body weight and food intake of the male and female rats in the model group were consistently lower than those in the control group. The body weight and food intake of male and female rats in the SSD group were consistently higher than those in the model group. HE staining was performed of the gastric sinus tissue for observation (Fig. **2C**). No organic changes were observed in the gastric tissues of the rats in any group. The tissues from the control and PC groups exhibited intact glandular structures and pinkish gastric mucosa. The gastric mucosa of the model group rats was whitish, and a small amount of inflammatory cell infiltration was visible on the surface of the glands. The SSD group showed substantial improvement in symptoms compared with the model group, with no notable inflammatory cell infiltration or normal gastric mucosal structures. As shown in Fig. (**2D**), the ultrastructure of the ICCs in the control and PC rats was clear, with more slit connections and tighter connections with the surrounding cells and nerve fiber endings. The number of ICCs in the model group was lower, the ICCs were smaller, and a large number of autophagic vesicles appeared. After the administration of SSD, the ICC ultrastructure markedly improved and was clear. The TUNEL assay revealed that compared with the control group, the model group had a higher number of apoptotic cells in the gastric antrum tissue. The number of apoptotic cells in the SSD group was significantly lower than that in the model group Fig. (**2E**). In addition, we detected the expression of the autophagy-related protein LC3 II/I in gastric sinus tissue *via* Western blotting (WB; Fig. **2F**). LC3 II/I protein expression was significantly upregulated in the model group compared with that in the control group (*P<*0.01). LC3 II/I protein expression was significantly downregulated in the SSD group compared with that in the model group (*P<*0.05). The propulsion rate in the small intestine was significantly lower in the model group than that in the control group (*P<*0.01). The propulsion rate in the small intestine of rats was significantly higher after SSD treatment (*P<*0.05; Fig. **2F**). These results illustrate that SSD can improve the pathological changes and ultrastructure of ICCs and inhibit excessive autophagy.

### 3.3 Construction of Autophagy Models for ICCs

**Fig. (3):**
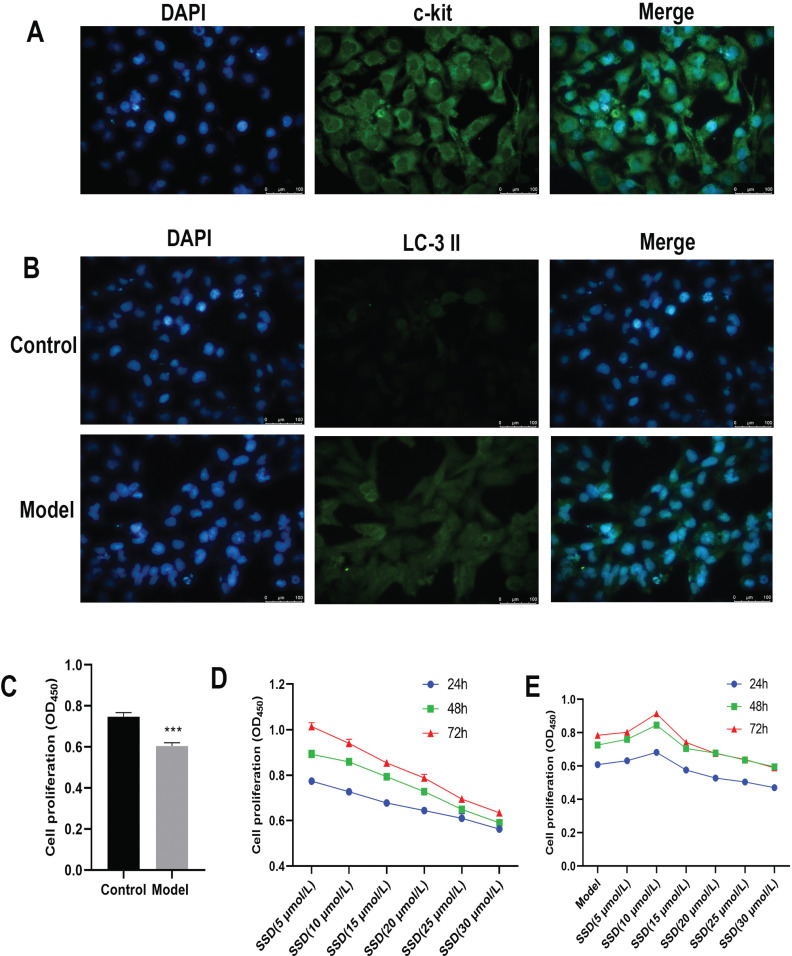
Construction of ICC autophagy model. (**A**) Identification of cell types *via* immunofluorescence staining using tyrosine protein kinase receptor c-kit monoclonal antibody. (**B**) Construction of ICC autophagy model *via* immunofluorescence detection of LC-3 II expression. CCK8 detection of (**C**) cell viability, (**D**) SSD toxicity, and (**E**) proliferation ability of each group of cells.

We then induced ICC autophagy using glutamate. The morphology of the cultured cells was observed under a microscope, and the cell types were identified *via* immunofluorescence staining using tyrosine protein kinase receptor c-kit monoclonal antibody. As shown in (Fig. **3A**), c-kit green fluorescence was observed. LC-3 II expression was detected using immunofluorescence (Fig. **3B**). Compared with the control group, the green fluorescence intensity of LC-3 II was substantially stronger in the model group. The proliferative power of the cells was determined using a CCK8 assay. The proliferative power of ICCs was significantly lower in the model group than in the control group (*P<*0.001; Fig. **3C**). This result indicated that an ICC autophagy model was successfully constructed. In addition, the toxicity of different concentrations of SSDs toward ICCs at different concentrations was determined using a CCK8 assay. As shown in Fig. (**3D**), the proliferative power of the ICCs gradually decreased after 24, 48, and 72 h of treatment with different concentrations of SSD. The optimal concentration and time of action of SSD were determined using a CCK8 assay. After SSD intervention for 24, 48, and 72 h, the proliferative power of the ICCs gradually increased with SD concentrations 5 and 10 μmol/L, which was followed by a gradual decrease in the proliferative power of ICCs. SSD at 10 μmol/L and 48 h was selected for subsequent experiments (Fig. **3E**).

### 3.4 Effect of SSD on Autophagy of ICCs

**Fig. (4):**
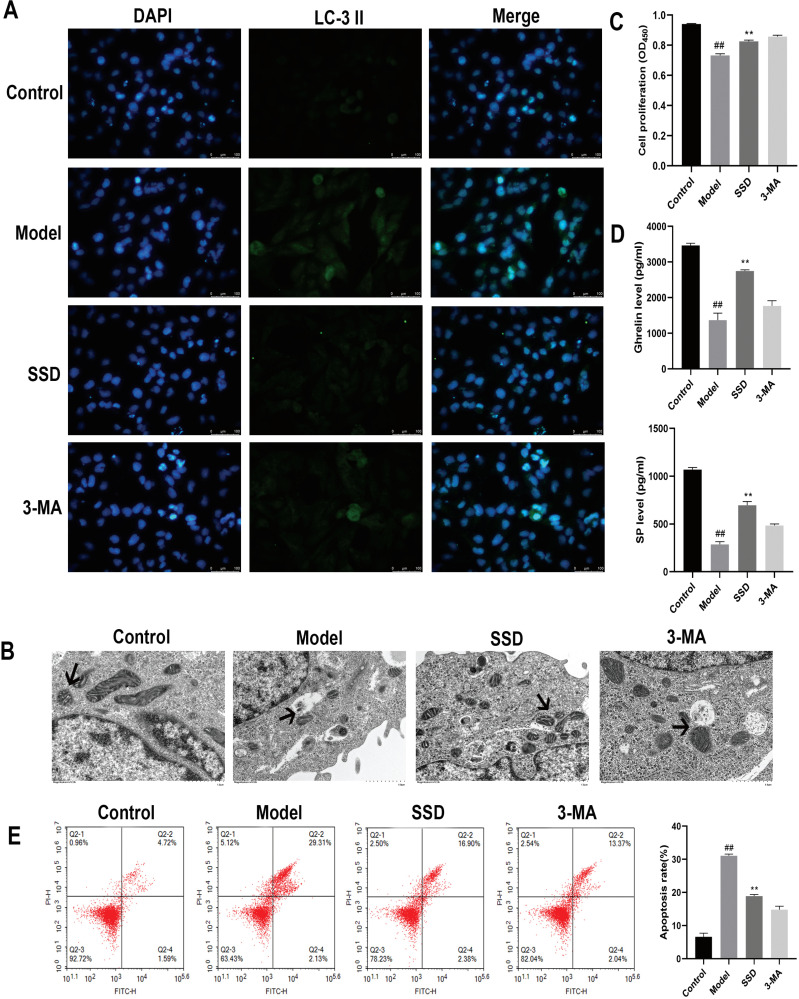
Effect of SSD on autophagy in ICCs. (**A**) Immunofluorescence assay conducted to detect LC-3 II expression. (**B**) Transmission electron microscopy conducted to observe autophagosomes and ultrastructure. (**C**) CCK8 assay used to detect cell proliferation. (**D**) ELISA results of ghrelin and SP expressions. (**E**) Flow cytometry results showing apoptosis rate.

The effect of SSD on the autophagy of ICCs was observed following drug intervention. The green fluorescence intensity of LC-3 II expression was strongest in the model group in the immunofluorescence assay and weakened after SSD intervention (Fig. **4A**). Autophagosomes and the ultrastructure of the ICCs were observed using transmission electron microscopy (Fig. **4B**). The ultrastructure of the ICCs of rats in the control and 3-MA groups was clear, and the ICCs were oval in shape with large nuclei. The nuclei of the ICCs in the model group were irregular in shape, with indistinct nuclear margins, increased heterochromatin, and a large number of autophagic vesicles. The situation considerably improved after SSD intervention, with substantially larger nuclei, a clear ultrastructure, and a notable reduction in the number of autophagic vesicles. The proliferative power of the cells in each group was determined using a CCK8 assay (Fig. **4C**). The proliferative power of the ICCs was significantly lower in the model group than in the control group (*P<*0.01). The proliferative power of the cells in the SSD group was significantly higher than that in the model group (*P<*0.01). ELISA was performed to detect the expression of factors regulating intestinal motility and secretion (Fig. **4D**). Compared with the control group, the expressions of both ghrelin and SP in the model group were significantly lower (*P<*0.01). The expression levels of both ghrelin and SP were significantly higher in the SSD group than in the model group (*P<*0.01). The rate of apoptosis was determined using flow cytometry (Fig. **4E**). The apoptotic rate of ICCs was significantly higher in the model group than in the control group (*P<*0.01) and significantly lower in the SSD group than in the model group (*P<*0.01). These results further suggest that SSD inhibits excessive autophagy and apoptosis in ICCs and promotes intestinal motility.

## 4 DISCUSSION

Currently, the available pharmacological treatments are limited and inadequate for improving the symptoms of patients with FD [24]. FD is a heterogeneous disease; therefore, a uniform response to drug therapy is impossible. In this study, the effect of the administration of SSD on the pathological changes and ultrastructure of ICCs was observed by constructing a rat model of FD. SSD administration improved the pathological changes and ultrastructure of ICCs and inhibited excessive autophagy. ICCs, a special type of intestinal cell, can generate spontaneous inward currents (called pacemaker currents) in the gastrointestinal tract to produce slow waves, which determine the frequency and timing of gastrointestinal smooth muscle contractions and play an important role in regulating gastrointestinal motility, including intestinal neurotransmitters and endogenous substances. Drugs regulate gastrointestinal motility by altering the frequency and structure of slow waves [25, 26]. Abnormal energy metabolism leads to the autophagy of ICCs, which results in reduced ICC activity and ultimately leads to gastrointestinal motility disorders, causing gastrointestinal diseases such as FD [25].

Gastrointestinal motility disorders are the main pathophysiological basis of FD. Gastric emptying refers to the passage of gastric content from the stomach into the duodenum and is used for assessing changes in gastric dynamics. After food and nutrients enter the gastrointestinal tract combined with mechanical and enzymatic chemical digestion, they become small, absorbable molecular substances and are used by the body to promote growth [27]. As such, digestive function is related to body weight, food intake, and food use [28]. FD is usually closely related to eating habits, and patients with FD are unable to complete normal digestive activities [29], which may be caused by abnormalities in gastrointestinal motility and gastric emptying. In this study, the rats in the model group had gastrointestinal motility disorders. The administration of SSD substantially increased the propulsion rate in the small intestine, promoted gastric emptying, and increased weight gain. The results obtained *via* light microscopy showed that the gastric sinus mucosa of all groups of rats was light red, with a smooth surface, clear structural levels, and intact gastric mucosal epithelium, and without notable swelling, ulceration or other organic lesions. These symptoms are consistent with nonorganic lesions in the FD rat model.

Autophagy participates in a wide range of processes in the pathogenesis of various diseases, including the lysosomal degradation of substrates such as long-lived proteins, aggregation-prone proteins, and damaged organelles, to maintain cellular homeostasis [30, 31]. LC3, a widely used autophagy marker, is involved in autophagic vesicle formation. LC3-II is a standard marker of autophagosomes that is produced by the binding of cytoplasmic LC3-I to phosphatidylethanolamine (PE) on the surface of nascent autophagosomes [32]. Zhang *et al.* found that FD promoted the production of autophagosomes in ICCs, increased the expression of the autophagy biomarkers Beclin1 and LC3, and decreased the expression of the differentiation biomarkers c-kit and SCF [33]. Our experiment was performed using a model of glutamate-induced autophagy in ICCs. The fluorescence intensity of LC3-II was substantially stronger in the ICCs of the model group, whereas the fluorescence intensity of LC3-II became notably weaker after SSD intervention. In addition, changes in the number, abnormal distribution and structural disruption of ICCs are key to the development of gastrointestinal motility disorders [23, 34]. A growing number of researchers have found that ICCs can be used as an important treatment for gastrointestinal motility disorders [35]. Ohlsson *et al.* found that in Crohn's disease, ICCs in the small intestine show atrophy and vacuolar degeneration, along with a decreased number [36]. Huizinga *et al.* studied ICCs in gastrointestinal motility and found that the ICC numbers were reduced or absent in the small intestine and colon, which do not exhibit normal peristaltic activity [37]. This is consistent with the results of the present study, in which the rats in the ICC autophagy model group showed a decrease in cell proliferation and an increase in the apoptotic rate, whereas after SSD intervention, the proliferation of ICCs cells was considerably higher and the apoptotic rate decreased. SSD administration increased the number of ICCs and improved gastrointestinal motility.

Ghrelin is a peptide hormone that is involved in gastrointestinal motility and secretion [38]. Ghrelin, an endogenous ligand for the growth hormone ghrelin receptor, is mainly produced by endocrine cells in the gastric-acid-secreting mucosa and has strong growth hormone-releasing activity [39, 40]. Kim *et al.* found that an herbal treatment (Banha-sasim-tang) improved FD by modulating ghrelin [41]. Hwang *et al.* found that yeokwisan enhanced gastric emptying by modulating the ghrelin pathway in a loperamide-induced FD mouse model [42]. SP and calcitonin gene-related peptide (CGRP) are involved in the sensitization of afferent neuronal pathways to chronic inflammation. In FD, the low perception thresholds for gastric dilatation are associated with high levels of CGRP and SP in the gastric sinuses, suggesting that sensory neuropeptides are involved in the pathophysiology of FD [43]. In this study, the expressions of ghrelin and SP decreased in the ICC autophagy model group, and the expressions of ghrelin and SP were elevated in ICCs after SSD treatment.

## CONCLUSION

We found that treatment with SSD improved the pathological morphology of ICCs and the morphological structure of gastric sinus ICCs, increased the protein expression of LC3 I/II, inhibited the excessive autophagy of ICCs, and promoted gastric emptying in an FD rat model. By constructing an autophagy model of ICCs, we further verified that SSD could improve the ultrastructure of ICCs cells, inhibit excessive autophagy and apoptosis, and promote intestinal motility. Moreover, SSD administration improved FD gastrointestinal motility disorders by modulating ghrelin and SP levels.

## Data Availability

The data used to support the findings of this study are available from the corresponding authors [H.Z. and Y.G.] upon request.

## LIST OF ABBREVIATIONS

CGRP: Calcitonin Gene-related Peptide
GHSRs: Growth Hormone Secretagogue Receptors
NO: Nitric Oxide
OD: Absorbance
PE: Phosphatidylethanolamine
SD: Standard Deviation
SP: Substance P
SSD: Saikosaponin D
